# Operationalizing the Consolidated Framework for Implementation Research to build and support the lived experience workforce in direct health service provision

**DOI:** 10.1111/hex.14035

**Published:** 2024-04-03

**Authors:** Alayna Carrandi, Yanan Hu, Katherine McGill, Sarah Wayland, Shae Karger, Myfanwy Maple

**Affiliations:** ^1^ Social Work, School of Health University of New England Armidale Australia; ^2^ School of Public Health and Preventive Medicine, Department of Epidemiology & Preventative Medicine Monash University Melbourne Australia; ^3^ Women's Health Economics and Value Based Care, Monash Centre for Health and Research and Implementation Monash University Clayton Australia; ^4^ School of Medicine and Public Health, College of Health, Medicine and Wellbeing University of Newcastle Newcastle Australia; ^5^ Healthy Minds, Hunter Medical Research Institute Newcastle Australia; ^6^ Mental Health‐Research, Evaluation and Dissemination (MH‐READ), Hunter New England Local Health District Newcastle Australia

**Keywords:** client participation, co‐creation, lived experience, patient involvement, peer, peer provision

## Abstract

**Background:**

The involvement of people with lived experience (LEX) workers in the development, design, and delivery of integrated health services seeks to improve service user engagement and health outcomes and reduce healthcare gaps. Yet, LEX workers report feeling undervalued and having limited influence on service delivery. There is a need for systematic improvements in how LEX workforces are engaged and supported to ensure the LEX workforce can fully contribute to integrated systems of care.

**Objective:**

This study aimed to operationalize the Consolidated Framework for Implementation Research (CFIR) using a rigorous scoping review methodology and co‐creation process, so it could be used by health services seeking to build and strengthen their LEX workforce.

**Search Strategy:**

A systematic literature search of four databases was undertaken to identify peer‐reviewed studies published between 2016 and 2022 providing evidence of the inclusion of LEX workers in direct health service provision.

**Data Extraction and Synthesis:**

A descriptive‐analytical method was used to map current evidence of LEX workers onto the CFIR. Then, co‐creation sessions with LEX workers (*n* = 4) and their counterparts—nonpeer workers (*n* = 2)—further clarified the structural policies and strategies that allow people with LEX to actively participate in the provision and enhancement of integrated health service delivery.

**Main Results:**

Essential components underpinning the successful integration of LEX roles included: the capacity to engage in a co‐creation process with individuals with LEX before the implementation of the role or intervention; and enhanced representation of LEX across organizational structures.

**Discussion and Conclusion:**

The adapted CFIR for LEX workers (CFIR‐LEX) that was developed as a result of this work clarifies contextual components that support the successful integration of LEX roles into the development, design, and delivery of integrated health services. Further work must be done to operationalize the framework in a local context and to better understand the ongoing application of the framework in a health setting.

**Patient or Public Contribution:**

People with LEX were involved in the operationalization of the CFIR, including contributing their expertise to the domain adaptations that were relevant to the LEX workforce.

## 1 INTRODUCTION

The rapid development of a lived experience (LEX) workforce in healthcare systems has been catalyzed by the evolution of international healthcare policies towards person‐centered, integrated systems of care and away from a traditional biomedical model.^1^, ^2^, ^3^ The current literature highlights that people who have direct experience of a health condition or health service—people with LEX—are best positioned to identify existing gaps and inform improvements to better meet service users' needs.^1^, ^3^, ^4^, ^5^ Introducing LEX workers into service delivery, therefore, is a key step to establishing integrated systems of care.

Acknowledging the multiple terms and definitions associated with LEX workers across a range of disciplines, this research will refer to all peer roles where a person is formally employed in a nonclinical capacity to provide direct support to peers and whose LEX of a health condition or service use is one of the main qualifications for employment^6^—hereafter referred to broadly as *LEX workers*. People with LEX informing this work are those for whom their LEX is at the forefront of their contribution, noting that they will have many other experiences and skills. People with LEX can exist at various organizational levels, including but not limited to administration, management, policy, research, and education. Furthermore, people with LEX can take on many different roles in an organization, including roles where LEX is not explicitly defined in their role description. For the purposes of this research, LEX workers provide a health service where both the target and agent of the service have LEX of the health condition or health service, meaning the activities that are performed by a LEX worker occur at a peer‐to‐peer level and avoid power differentials. *Nonpeer staff* refers to administrative (i.e., nonclinical) and clinical staff in a health setting of which LEX is not a qualification of employment. *Service users* are people who access a health service (i.e., patients), and *carers* are people who care for or accompany service users.

The growing number of roles undertaken by those with LEX in health services has been identified as an essential and valuable component of service delivery.^7^ This trend has not been without challenges, including a lack of role clarity and professional credibility identified by both LEX workers and their counterparts (e.g., health professionals and administrative staff).^3^, ^8^ Policies and frameworks have been developed to support the employment and training of people with LEX across systems of care.^9^, ^10^, ^11^ Other local frameworks exist in the grey literature, which focus on guiding the process of implementation (process frameworks). Still, there is a need for a systematic approach to understand how to develop and support the introduction and growth of the LEX workforce.^3^, ^7^, ^11^, ^12^, ^13^, ^14^, ^15^ The systematic development of a determinant implementation framework is necessary to guide how the LEX workforce can be sustained across healthcare systems. In turn, this offers service providers the opportunity to expand the LEX workforce and deliver more integrated care.

Determinant implementation frameworks, such as the Consolidated Framework for Implementation Research (CFIR), can be used by service providers to plan and inform real‐world implementation efforts.^16^ The purpose of the CFIR is to clarify terminology and assess the extent to which the implementation of an innovation is effective across a range of settings and contexts.^17^ The domains of the CFIR include the contextual components of the innovation—the intervention characteristics, inner and outer setting, and the characteristics of the people involved—and the process by which implementation is accomplished.^17^, ^18^ As LEX roles continue to integrate into health service delivery and expand to other areas of health care, a determinant implementation framework can provide a systematic way to approach the introduction of LEX roles within health service provision.

Implementation science frameworks have been applied to discrete program evaluations but have not been used to inform implementation efforts across systems of care. Adaptations to the CFIR, such as using contextually relevant language, are essential to increase the applicability and usability of the framework and to fully operationalize the constructs within organizations wishing to better support and build a LEX workforce.^18^ Further, although co‐creation processes have not been previously used to operationalize implementation science frameworks, co‐creation aligns with the values underpinning LEX by encouraging the participation of people with LEX to influence healthcare services.^19^, ^20^ This study aimed to operationalize the CFIR using a rigorous scoping review methodology and co‐creation process, so it could be used by health services seeking to build and strengthen their LEX workforce. This process will generate actions organizations can take to ensure an effective approach to engaging a LEX workforce. Operationalizing the CFIR framework will, therefore, focus on the contextual components that support a successful LEX *role*, rather than evaluating a LEX *worker* whose value may be tainted by a lack of supportive organizational structures and policies.

## 2 MATERIALS AND METHODS

**Figure 1:**
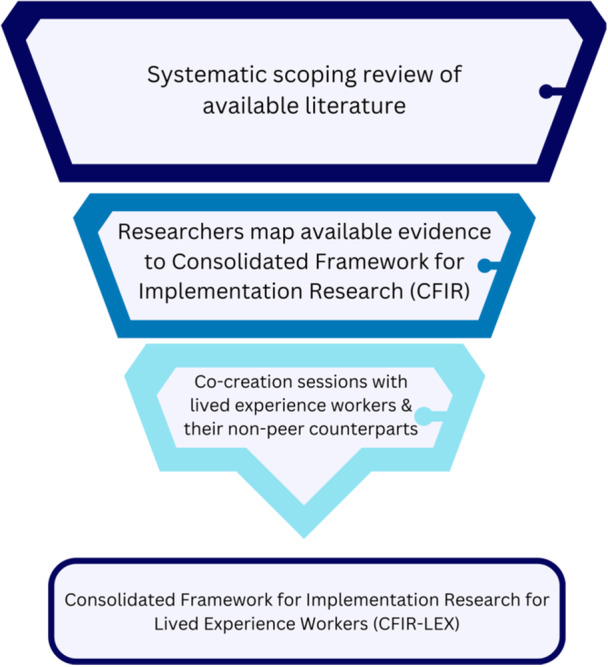
Scoping review methodology and co‐creation process used to develop and refine the implementation framework of lived experience workers in direct health service provision.

A multifaceted approach was used to develop and refine the implementation framework of LEX workers in direct health service provision, including (1) a systematic scoping review of available literature, (2) the development of a preliminary framework, and (3) co‐creation sessions with LEX workers and their nonpeer counterparts to refine the framework. This process followed Arksey and O'Malley's five‐step scoping review framework and the optional sixth step of a consultation exercise.^21^ We used the findings from the scoping review to develop a preliminary framework, which informed the co‐creation sessions as recommended by Levac et al.^22^ The purpose of the co‐creation sessions was to validate the findings from the scoping review, build on available evidence, and offer a higher level of meaning, context expertise, and perspective on the findings. A similar method has been previously used to develop a testable conceptual framework to prospectively and iteratively identify the likely implementability of healthcare interventions.^23^ The process is further illustrated in Figure 1.

### 2.1 Scoping review

A scoping review was chosen because it is best suited to map key concepts across the evidence base.^21^ The research question guiding the scoping review was: ‘How are lived experience workers effectively involved in health service provision?’. A search strategy was developed in partnership with a university librarian and then adapted for each of the included databases: PsychINFO, PsychArticles, Scopus, and Informit (Supporting Information S1: Appendix 1). A systematic literature search of databases was undertaken on 16 July 2021 and 27 September 2022 to identify peer‐reviewed studies providing evidence of the effectiveness of involving LEX workers and their roles and expectations in health service provision (Table 1). Research focused on informal support (e.g., peer support groups or online networks) and nondirect support (e.g., administration, research, or formal education) was excluded. Studies that did not explicitly state the worker had LEX were also excluded. Due to the emerging and rapidly changing nature of the health workforce, the search was limited to the years 2016–2022. This period covered the time since the development of international guidelines and frameworks for peer support workers.^24^, ^25^, ^26^, ^27^ Two independent reviewers (A. C., Y. H., S. K.) screened titles and abstracts, then full‐text articles in the Covidence platform.^28^ Disagreements that arose between the reviewers during screening were resolved by a third reviewer (A. C., Y. H., S. K.). Reviewers met after each stage in the screening process and engaged in a reflexive discussion, as the terms and definitions used to describe LEX workers varied extensively. Two reviewers (A. C., Y. H.) independently extracted data from each study included in the review and examined the other reviewers' data extraction process. Reviewers met intermittently throughout the data extraction process to modify and revise the tool as necessary. The finalized data extraction form is presented in Supporting Information S1: Appendix 2.

**Table 1:** Inclusion and exclusion criteria.

Inclusion criteria	Exclusion criteria
An individual was employed (paid/unpaid) to provide direct support to service users	People with lived experience were involved in providing education, conducting research, or informing policy
Described the effectiveness of involving lived experience workers or the experiences of lived experience workers in health service provision from the perspective of patients or service users, other health professionals and administrators, stakeholders, and lived experience workers themselves	Studies that focused on patient outcomes (e.g., effectiveness) as a result of incorporating lived experience roles
Lived experience workers applied experiential knowledge to support service users	Informal support where people shared personal stories in a peer‐to‐peer capacity (e.g., peer support groups or online networks)
Lived experience of a health condition and/or service use was one of the main qualifications for employment in the lived experience role	Person providing service did not have lived experience The person receiving service did not have lived experience
Peer‐reviewed primary studies	Secondary or opinion sources (e.g., systematic reviews and editorials)
Published in English after 2016	Non‐English study published before 2016

### 2.2 Development of the preliminary framework

We used the CFIR as a scaffold for the development of the framework to guide efforts to build and strengthen the LEX workforce in health service provision.^17^, ^18^ The descriptive‐analytical method was used to map findings from the scoping review to the relevant domains and constructs of the CFIR based on the descriptors for each domain and construct. Each study was assessed by one reviewer (A. C.) to determine whether the study reported on each domain and construct of the CFIR. Relevant study results were compiled into each domain and construct and then summarized. Another reviewer (M. M.) verified these assessments with disagreements resolved through discussion.

### 2.3 Co‐creation engagement sessions

Two co‐creation focus group sessions were conducted in October 2022. The purpose of these sessions was to validate findings from the available literature and to identify other key concepts in the development and growth of a LEX role. The first co‐creation session was held with four LEX workers, and the second session was held with two people who work alongside LEX workers (‘non‐peer counterparts’). Ethical approval was granted by the University of New England's Human Research Ethics Committee (Approval No. HE21‐224).

LEX workers and their nonpeer counterparts were recruited from a state‐wide suicide postvention service in Australia. The research team discussed the framework development process with the service's leadership team, including those with LEX, throughout the project. Workers were invited through internal networks to participate in a 1‐h session via videoconferencing (Zoom). These workers were selected for the focus group because of their unique roles in skilled LEX positions. A mutually suitable time was decided upon, and people interested in participating were sent an email containing a description of the project alongside the preliminary framework 1 week before the co‐creation session. The co‐creation sessions involved semistructured discussions about their experiences in their roles and critical aspects of the implementation process. No demographic information was collected. During the co‐creation session with LEX workers, discussions centered around the final domain of the CFIR, the implementation process, because there was no evidence of this domain in the academic literature. Discussions with nonpeer counterparts focused on the inner setting (Domain Three), including structural characteristics, relational connections, communications and implementation culture, and the individuals domain (Domain Four), including others who have supported or hindered the involvement of LEX workers in the organization. We utilized an online software (Miro) during the session to visually collaborate, share ideas, and engage with one another. Following the co‐creation sessions, the preliminary framework was revised and sent to LEX workers and their nonpeer counterparts via email. They had the opportunity to review the revised framework and provide any final comments until a consensus was reached.

## 3 RESULTS

### 3.1 Scoping review

The systematic search retrieved 1002 studies. With 567 duplicates removed, 434 titles and abstracts were screened. A total of 166 full‐text articles were screened, and 72 studies met the inclusion criteria for the purposes of this review. Among the studies identified, three‐quarters (76%) were in the mental health setting. The other identified studies were in behavioral health (e.g., substance use or infectious diseases), chronic disease (e.g., diabetes or multiple sclerosis), and community health (e.g., homelessness or criminal justice). Most studies were conducted in high‐income countries such as the United States (38%), Australia (24%), and European countries (17%). A diverse range of terms was used to describe LEX workers, of which the most frequent were *peer worker* (35%) and *peer specialist* (19%). The definitions used to define a LEX worker included the following components: (a) provides support; (b) has LEX; (c) has completed formal training; (d) embodies the values of LEX and (e) works within a team. Most studies (*n* = 57, 79%) qualitatively explored the integration of LEX workers into a health setting and the associated challenges, benefits, roles, and expectations. Among studies that reported quantitatively on the effectiveness of LEX roles (*n* = 15, 21%), over half (*n* = 8) reported statistically significant positive health outcomes associated with the involvement of a LEX worker.

### 3.2 Development of the preliminary framework

The terms and descriptions of the original CFIR domains were adapted for the context of building and strengthening the LEX workforce in health service provision using the findings from the scoping review. The original CFIR has six domains, and the identified studies from the scoping review reported on four domains. There was no available evidence for the individuals domain (Domain Four) and the implementation process domain (Domain Six). Supporting Information S1: Appendix 3 provides a narrative summary of the literature relevant to each domain. Key aspects of the innovation domain (Domain One) covered in the literature included the value of having a LEX workforce from the perspectives of service users, nonpeer staff, and LEX workers themselves. The literature related to the outer setting (Domain Two) covered the challenges associated with external policies governing and formalizing LEX workforces. The organizational structures that promote and hinder the effective integration of LEX workers, including supervision and organizational representation of LEX, were covered in the literature related to the inner setting (Domain Three). The literature related to the characteristics of LEX workers (Domain Five) described the core and peripheral components of a LEX worker's role.

### 3.3 Co‐creation process

The LEX workers (*n* = 4) described the value of being involved during the entire implementation process, but some workers said they wished they had been more involved earlier in creating their role. They expressed the importance of differentiating their role from other roles and the additional training required to fulfill their role. The LEX workers valued the presence of LEX across the organizational structure and the ability to link with other LEX workers to debrief and process their experience in the role. They described their role as dynamic and valued the pay increase, training opportunities, and supervision they received when their duties expanded throughout the implementation period. Being adequately remunerated was key to feeling valued as a staff member. LEX workers discussed the issues of retention, turnover, and being understaffed, and attributed this to the intensity of the role, highlighting the importance of boundaries and self‐care.

LEX workers expressed that service users did not always understand their role and sometimes expected a clinical solution. At times, the service users perceived the LEX workers as volunteers, so they may have felt like they were burdening the LEX workers. The LEX workers attributed the misunderstanding to stigma and undervaluing LEX. However, the LEX workers did believe that service users appreciated them and the sense of validation and hope they received. Being able to give feedback to the organization about their experience in the role and what the service users need was important to LEX workers. Different feedback methods included monthly check‐ins with a peer mentor through an external organization, recurring meetings with managers, and optional debriefs following a session with service users. Some LEX workers found various modes of feedback helpful. Overall, providing feedback and sensing that the feedback was taken on board was critically important to the LEX workers.

Topics discussed with nonpeer counterparts (*n* = 2) included the distinct value of LEX to service provision and needing a framework to guide the development and growth of a LEX workforce. Nonpeer counterparts described difficulties recruiting a LEX worker due, in part, to the stigma that exists towards people with LEX in the community. Similar to the LEX workers, they also discussed the importance of having people with LEX at various levels within an organization and having training available for those providing support to LEX workers. Nonpeer counterparts also identified some of the key individuals involved in the successful implementation of the LEX workforce. Findings from the co‐creation sessions are further described in Supporting Information S1: Appendix 3.

### 3.4 Fully operationalized CFIR for the implementation of a LEX workforce

Using the information from the co‐creation sessions with the findings from the scoping review, we operationalized the CFIR‐LEX framework. This adapted CFIR provides guidance about the structural policies and strategies that allow people with LEX to actively participate in the provision and enhancement of integrated service delivery across all domains. The CFIR‐LEX domains one through five are described with short definitions and operationalizing questions in Table 2. These domains include the following: the characteristics of the innovation being implemented (Domain One); external economic, political, and social context (Domain Two); internal structural, political, and cultural context (Domain Three); and roles and characteristics of those involved in the implementation process (Domains Four and Five). The implementation process is the sixth and final domain of the CFIR and consists of the stages of the implementation of an innovation in an organization, or the process by which implementation is accomplished. The operationalizing questions are presented to assist those using the framework to consider relevant aspects under each domain. This implementation framework can be used as a determinant framework and can be used alongside other frameworks for context‐specific guidance.^16^

**Table 2:** Operationalized CFIR for the implementation of a lived experience workforce—quick reference.

CFIR‐lived experience domains and constructs	Key points	Operationalizing questions
Domain One: Innovation domain *Document the innovation being implemented and distinguish the innovation from the implementation process*.		
Construct 1: Innovation source *External or internal development of the lived experience role*	Lived experience workers are engaged in the process of defining the scope of their role and in the development of interventions delivered, to meet the needs of service users in a local context.	Are lived experience workers engaged in the hiring and training process? Are lived experience workers defining the scope of their role? Are lived experience workers designing the intervention?
Construct 2: Innovation evidence‐base *Quality and validity of evidence supporting the effectiveness of lived experience in health service provision*	The lived experience role has robust evidence supporting its effectiveness in health service delivery.	Has available evidence been reviewed regarding the use of a lived experience role in the current context?
Construct 3: Innovation relative advantage *Stakeholders’ perceptions of the advantage of implementing a lived experience role versus treatment as usual or using a nonpeer staff member*	Lived experience is complementary to the traditional health service delivery model. Lived experience workers also add a distinct value to service provision by focusing on relationship‐building and role‐modelling.	Do stakeholders perceive the lived experience workers role as an addition to the current service delivery model? Do stakeholders perceive the value of having a lived experience worker role deliver the intervention, as opposed to a nonpeer staff member? Is this value clearly conveyed to the service user? Do stakeholders understand the role of a lived experience worker in the context of a traditional health service?
Construct 4: Innovation adaptability *The degree to which the lived experience role can be adapted, tailored, refined, or reinvented to meet the needs of the organization and service users*	Engaging lived experience workers in generative activities allows them to tailor the service to meet the unique needs of the service user and fit the local context.	Is the role of the lived experience worker flexible? Is the role of the lived experience worker and the activities delivered by the lived experience worker able to be adapted to suit local needs and service users?
Construct 5: Trialability *The ability to pilot the lived experience role in the organization and be able to reverse course if warranted*	Piloting a lived experience workforce on a small scale in an organization allows for ongoing, constructive feedback between the lived experience worker and their managers and supervisors. The pilot phase must allow the role responsibilities to change, the position description to be refined, and training to be provided, when necessary.	What feedback systems are in place to allow new lived experience workers to provide ongoing, constructive feedback during the pilot period? Which evidence‐based models or frameworks are being used to guide the implementation of a lived experience role?
Construct 6: Innovation complexity *Perceived difficulty in introducing a lived experience role with respect to the scope and number of steps required to implement*	Complexities arise when lived experience workers are not engaged at an earlier stage in the development process, confusing a lived experience workers' role and credibility. The introduction of lived experience workers at an earlier stage in the development of the role and intervention is a preventative way of wasting resources or experiencing delays due to unforeseen barriers.	Are the role competencies and outcome measures developed in such a way as to ensure the integration of the lived experience role is successful? Does a system of active and consistent communication exist between lived experience workers and nonpeer staff during the stage of implementation to avoid confusion in the value and role of the lived experience worker? Does the lived experience worker understand their role in the service model? Does the nonpeer staff understand the role of the lived experience worker in the service model?
Construct 7: Innovation design *Perceived excellence in how a lived experience worker delivers an intervention*	Clear communication with service users and nonpeer staff on what to expect from services delivered by a lived experience can optimize the lived experience workers' experiences delivering the service and improve the service users’ experiences with the service. Further, linking lived experience workers with other lived experience workers can help them integrate into the service and have clearer expectations for the role.	Are we clearly communicating with service users and nonpeer staff on the role of a lived experience worker within our service model? Are there opportunities for lived experience workers to connect with one another to achieve clearer expectations for their role?
Construct 8: Innovation cost *Costs of introducing a lived experience worker*	The financial remuneration of lived experience workers must be discussed during the hiring process to prevent job dissatisfaction. Lived experience workers also require ongoing support and training which must receive adequate resource allocation upon implementation.	Will the lived experience worker be a staff or volunteer? What is the cost of ongoing support and training required to support a lived experience worker? Are lived experience workers adequately remunerated in accordance with their position description?
Domain Two: Outer setting domain *The outer setting refers to the economic, political, and social context within which an organization resides*.		
Construct 1: Critical incidents *Large‐scale and/or unanticipated events disrupt the implementation of the lived experience worker*	Implementation efforts may be disrupted, so organizations must develop a critical incident response plan.	Has a critical incident response plan been established?
Construct 2: Local attitudes *Sociocultural values* (*e.g., the shared responsibility of helping service users*) *and beliefs* (*e.g., convictions about the worthiness of service users*) *encourage the outer setting* (*e.g., community, system, state*) *to support the implementation of the lived experience worker*	Considerations must be made in the local context regarding the value placed on lived experience in direct health service provision.	What are the community attitudes towards lived experience? Is there a stigma towards the implementation of lived experience in direct health service provision?
Construct 3: Local conditions *Economic, environmental, political and/or technological conditions enable the outer setting to support the implementation of the lived experience worker*	Organizations may have to access external support for the implementation of the lived experience worker.	Are there external resources available to support the implementation of a lived experience workforce?
Construct 4: Partnerships and connections *The degree to which an organization is networked with other external organizations*	Lived experience workers can be sourced from outside organizations that provide peer services and train workers with lived experience. Connecting with peer organizations can facilitate relevant and valuable training to lived experience workers.	From where will the organization hire, or recruit lived experience workers? Are there existing peer organizations that can facilitate relevant and valuable training to our lived experience workforce?
Construct 5: Policies and laws *External policy and regulations* (*e.g., external mandates, recommendations and guidelines, pay‐for‐performance, collaboratives, and public or benchmark reporting*) *pertaining to lived experience workers*	Lived experience workers have been introduced in a wide range of clinical and community settings. This may place pressure on organizations to implement a lived experience workforce and policymakers to regulate and fund the workforce and the services they provide.	What external policies exist to regulate and incentivize lived experience workers in health service delivery? How will external policy affect the integration of a lived experience worker into the service model?
Construct 6: Financing *Funding from external entities* (*e.g., grants, reimbursements*) *available to build and strengthen the lived experience workforce*	External funding may be available to drive the implementation of a lived experience workforce.	Is funding available from external entities to assist in the implementation of a lived experience workforce?
Construct 7: External pressure *Societal, market, and/or performance‐measurement pressures that drive implementation of a lived experience worker*	Societal pressures, such as mass media campaigns, advocacy groups or social movements, may place pressure on organizations to implement a lived experience workforce. Further, other peer or competing organizations, as well as quality or benchmarking metrics in the broader health system, may pressure organizations to implement a lived experience workforce.	Are there mass media campaigns, advocacy groups, or social movements driving the implementation of a lived experience worker in direct health service provision? Are there peer or competing organizations that have already implemented a lived experience workforce? Are there quality or benchmarking metrics or established service goals driving the implementation of a lived experience worker in direct health service provision?
Domain Three: Inner setting domain *The inner setting includes features of structural, political, and cultural contexts through which the implementation process will proceed*.		
Construct 1: Structural characteristics *Infrastructure components that support functional performance of the inner setting*	The presence of lived experience roles at other levels within the organization can overcome implementation challenges associated with the disconnect between people providing the service and those in leadership positions. Further clarification regarding role allocation between lived experience workers and nonpeer workers may be required. Considerations must also be made regarding the physical and information technology infrastructure required to implement a lived experience workforce.	Does lived experience exist at other nondirect levels within the organization? Is the organization of tasks and responsibilities within and between individuals and teams, and general staffing levels, clear and optimal to support the implementation of a lived experience workforce? Are the physical and information technology infrastructure adequate to support the implementation of a lived experience role?
Construct 2: Relational connections *The nature and quality of social networks within an organization* (*e.g., peer‐to‐peer and peer to nonpeer*)	A collaborative approach to supervision and support promotes a positive culture of communication and effective management of risk in health service provision. Supervision can be a way of providing practical support and mentorship to lived experience workers, as well as ensuring the roles and expectations of lived experience workers are agreed upon and clearly set out. By identifying a structured supervision model, training can be established to upskill people in providing support to lived experience workers.	What will be the nature of supervision for lived experience workers? Who will be providing supervision to lived experience workers? What skills are necessary for people providing support to lived experience workers? Which existing role is best suited to provide support to lived experience workers? If a role does not exist, is it possible to establish a new role?
Construct 3: Communications *The nature and quality of formal and informal communications within an organization* (*e.g., peer‐to‐peer and peer to nonpeer*)	The integration of lived experience into an organization requires the encouraged participation of lived experience workers in collaborative processes. Organizational strategies must enable lived experience workers to provide genuine consultation and transform approaches to service delivery.	Does the organization encourage participation of lived experience workers in collaborative processes? Do organizational strategies exist to enable lived experience workers to influence service delivery? Will lived experience workers be able to provide genuine consultation in the delivery of services within the organization?
Construct 4: Culture *Norms, values, and basic assumptions of an organization and how these align with the foundational values of lived experience*	An effective organizational culture for building and strengthening the lived experience workforce is one in which (1) leaders express the value and need for lived experience in their organization, (2) lived experience workers feel they are essential, valued, and knowledgeable partners in the change process, (3) lived experience workers feel psychologically safe in applying their expertise, and (4) there is sufficient time and space for reflective thinking and evaluation. One of the key challenges to the expansion of the lived experience workforce and its integration into an interdisciplinary team is the structural and interpersonal stigma that exists towards lived experience in nonrecovery‐oriented workplaces.	What stigma exists among nonpeer staff towards lived experience? What stigma exists within structural policies towards lived experience workers? How will the organization identify and address structural and interpersonal stigma?
Construct 5: Tensions for change *The degree to which stakeholders perceive the current situation* (*without lived experience workers*) *as needing change*	The uncertainty of stakeholders on the perceived value and role of lived experience workers negatively impacts the motivation to employ people with lived experience.	Do stakeholders perceive the current service delivery model (without lived experienced workers) as needing change?
Construct 6: Compatibility (1) *The degree of tangible fit between the roles and values attached to lived experience workers*, (2) *how those align with nonpeer professionals' norms, values, and perceived risks and needs, and* (3) *how lived experience workers fit in with existing workflows and systems*	The roles of a lived experience worker are distinct from other nonpeer roles and can add value to an interdisciplinary team. Compatibility relies on cooperation between lived experience workers and nonpeer workers.	Will lived experience workers be a part of an interdisciplinary team? Which facilitators are in place to allow for cooperation between lived experience workers and nonpeer staff?
Construct 7: Relative priority *Stakeholders’ shared perception of the importance of the implementation of lived experience within the organization*	Although regarded as largely positive, clarity on the role and value of lived experience workers varies among stakeholders. Nonpeer staff must consult and collaborate with lived experience workers to design their rand facilitate effective programs.	How do stakeholders perceive the necessity of lived experience workers to improve service delivery?
Construct 8: Incentive systems *Extrinsic incentives* (*e.g., goal‐sharing awards, performance reviews, promotions, and raises in salary*) *and less tangible incentives* (*e.g., increased stature and respect*)	Positive workplace experiences for lived experience workers include the following: job security, supervision and support, rewards and recognition, professional advancement, and adequate remuneration.	Does the organization provide incentives for lived experience workers, such as professional advancement, rewards, and recognition?
Construct 9: Mission alignment *The degree to which goals are clearly communicated, acted upon, and fed back to lived experience workers and the alignment of that feedback with goals*	Clear goals and feedback assist in clarifying the role and reinstating the value that lived experience workers provide in service provision.	Are goals clearly communicated to lived experience workers? Does the feedback received by lived experience workers reflect their role competencies?
Construct 10: Available resources *The level of resources dedicated to implementation and ongoing operations of lived experience workers* (*e.g., money, training, education, physical space, and time*)	The implementation of a lived experience workforce requires financial investment in training and remuneration packages, time spent building support structures, and ongoing evaluation and reflection.	Do we have the available resources to invest in training and remuneration for lived experience workers? Do we have the time to enact changes in current service delivery and support structures? Do we have the available resources and time to adequately evaluate and reflect on how to optimize the integration of lived experience workers into the organization?
Construct 11: Access to knowledge and information *Ease of access to digestible information and knowledge about the value and roles of lived experience workers and how to integrate lived experience workers into the service delivery model*	Guidance and/or training is accessible to lived experience workers and nonpeer staff to support the implementation of a lived experience role. Organizations that are not ordinarily recovery‐oriented, such as medical and clinical‐focused organizations, may require additional training for nonpeer workers in the values that underpin lived experience.	Does the lived experience worker have access to adequate and relevant training? What training is required for nonpeer staff to understand the value and role of lived experience workers in health service delivery?
Domain Four: Individuals domain *The roles and characteristics of individuals involved in the implementation process*.		
Construct 1: High‐level leaders *Individuals with a high level of authority, including key decision‐makers, executive leaders, or directors*	Commitment, involvement, and accountability of leaders and managers are essential in the effective integration of a lived experience workforce. Leaders and managers must be willing to implement and invest in changes to systems and processes to integrate lived experience workers into the service model. They must be responsive to feedback from lived experience workers and provide additional support and training, when necessary.	What systems are in place to integrate lived experience workers' feedback into the service model and existing processes? Are leaders and managers willing to be responsive to the feedback from lived experience workers? Is the organization committed to investing in the implementation of necessary changes to systems and processes to integrate lived experience workers into the service model?
Construct 2: Mid‐level leaders *Individuals with a moderate level of authority, including leaders supervised by a high‐level leader and who supervise others*	Nonpeer staff preparation and clear policies must exist to provide adequate resource allocation and ongoing technical support as the transition occurs.	Do those who oversee other workers or oversee service delivery (e.g., managers) value the integration of a lived experience workforce? What policies exist before the implementation of lived experience workers to ensure adequate resource allocation and ongoing technical support for nonpeer staff and lived experience workers? Has a model or framework been chosen to guide the implementation process? Are lived experience workers involved in the designing and developing of the intervention or service delivery model?
Construct 3: Opinion leaders *Individuals with informal influence on the attitudes and behaviors of others*	Opinion leaders may be people in lived experience or nonpeer roles.	Who are the opinion leaders in the organization? Are they able to influence the attitudes and beliefs of their colleagues?
Construct 4: Implementation facilitators *Individuals with subject matter expertise who assist, coach, or support implementation*	Identifying individuals within an organization, or hiring individuals, with expertise in lived experience implementation may be useful in the effective integration of a lived experience workforce.	Are there individuals within the organization with expertise in the implementation of a lived experience workforce? Is it feasible to hire someone with expertise in the implementation of a lived experience workforce to drive the implementation process?
Construct 5: Implementation leads *Individuals who lead efforts to implement the innovation*	Implementation leads may be people in lived experience or nonpeer roles.	Who is a formally appointed internal implementation leader? Do the internal implementation leaders formally appointed with responsibility for implementing a lived experience workforce and overseeing their work value the integration of a lived experience workforce?
Construct 6: Implementation team members *Individuals who collaborate with and support the implementation leads to implement the innovation, ideally including innovation deliverers and recipients*	Implementation team members may be people in lived experience or nonpeer roles.	Do the individuals who are dedicated to supporting the implementation of the lived experience workforce value the integration of a lived experience workforce?
Construct 7: Other implementation support *Individuals who support the implementation leads and/or implementation team members to implement the innovation*	External change agents may exist outside of the organization and formally influence decisions surrounding the implementation of lived experience workers.	What other adjacent organizations have implemented a lived experience workforce? How can we partner with these organizations and external change agents to support and expand the lived experience workforce?
Construct 8: Innovation deliverers *Individuals who are directly or indirectly delivering the innovation*	The core component of a lived experience workers' role is direct support to service users. Periphery components can include indirect support such as managerial roles, teaching skills, and systems‐level advocacy.	Are lived experience workers' core competencies to deliver direct services? What are the periphery components of the lived experience workers' role?
Construct 9: Innovation recipients *Individuals who are directly or indirectly receiving the innovation*	From a service user and carer perspective, services provided by lived experience workers are acceptable and add value to their care.	Will the needs of the service user be accurately addressed by introducing a lived experience worker in the service delivery model? How will the lived experience role be communicated to the service user and distinguished from a nonpeer role?
Domain Five: Characteristics of lived experience workers domain *The dynamic interplay between lived experience workers and the organization within which they work and how that interplay influences individual or organizational behavior change*.		
Construct 1: Attitudes *Lived experience workers' attitudes towards and value placed on their work*	The perceived value that lived experience workers add to service provision fuels their own recovery journey. Lived experience workers can also perceive the value of their work to the organization through the remuneration they receive.	Does the lived experience worker believe their work adds value to the service model? Does the lived experience worker believe their work fuels their own recovery journey? How do we indicate to the lived experience worker their value to the organization?
Construct 2: Self‐efficacy *Lived experience workers' belief in their capabilities to execute courses of action to achieve goals*	The lived experience worker's role can be intense and requires boundaries and a self‐care routine.	Does the lived experience worker have the necessary tools to establish boundaries in their role and a self‐care routine?
Construct 3: Self‐disclosure *Construction of a positive identity through self‐disclosure of lived experience as the lived experience worker progresses towards skilled, enthusiastic, and sustained use of lived experience*	Constructing a positive identity through self‐disclosure is a cornerstone of the lived experience role. This must be an ongoing process and can be constructed through training.	Does the lived experience worker engage in self‐disclosure? Is the lived experience worker able to construct a positive identity through self‐disclosure of their lived experience? What trainings exist to support lived experience workers in constructing a positive identify through self‐disclosure?
Construct 4: Boundaries *A broad construct related to how lived experience workers perceive themselves outside of their role and their ability to set boundaries between their personal recovery and others' recovery journey*	Lived experience workers must also create an identity outside of their lived experience and set boundaries with service users.	Is the lived experience worker able to set boundaries between their personal recovery and their role? Is the lived experience worker able to set boundaries between themselves and the service users?

Abbreviation: CFIR, Consolidated Framework for Implementation Research.

### 3.5 Key themes

Based on the findings from the scoping review, mapping available evidence to the CFIR domains and co‐creation sessions, the key components to the successful integration of a LEX worker in a health setting were identified as (1) an organizational commitment to engaging people with LEX in the co‐creation of the LEX role and how interventions could be delivered; and (2) the need for representation of people with LEX within an organization. These components were key themes identified by the authors across the various stages of the framework development process.

### 3.6 Engaging people with LEX in the co‐creation process

This research identified that although LEX workers were more commonly introduced later in the implementation process, the engagement of people with LEX in the earlier stages of implementation contributed to more positive outcomes for the LEX workers concerning job satisfaction and an increased sense of purpose.^29^, ^30^, ^31^ The importance of engaging LEX workers in the process of defining the scope of their role and in the development of interventions delivered are included in the innovation domain (Domain One) (Table 2). Positive outcomes were also cited for service users and nonpeer staff regarding a more person‐centered environment.^32^, ^33^, ^34^, ^35^, ^36^, ^37^, ^38^, ^39^, ^40^, ^41^, ^42^, ^43^ In the co‐creation sessions, nonpeer workers discussed difficulties filling the LEX role in certain areas due to local attitudes and stigma towards identifying as someone with LEX. Nonpeer workers found that partnering with external organizations whose focus is training and supporting LEX workers was helpful in the implementation process. Considerations of the external economic, political, and social context within which an organization resides are included in the outer setting domain (Domain Two) (Table 2). Although the implementation process was not explicitly identified in the evidence review, the importance of this was discussed during the co‐creation sessions. To improve the translation of new knowledge into practice, optimize the implementation of health interventions, and enhance outcomes, the implementation process in the context of LEX workers can be mapped onto the four processes of co‐creation: co‐ideation, co‐design, co‐implementation, and co‐evaluation.^44^ Table 3 demonstrates where the co‐creation process can be mapped onto the implementation process domain (Domain Six) to ensure engagement with and representation of LEX throughout the collaborative processes.

**Table 3:** Operationalized CFIR for the implementation of a lived experience workforce domain six mapped to the co‐creation process.

CFIR‐lived experience domains and constructs	Key points	Operationalizing examples
Domain Six: Implementation process domain *The essential activities and strategies used to implement a lived experience workforce*.		
Construct 1: Teaming *The degree to which lived experience and nonpeer workers join together, intentionally coordinating and collaborating on the implementation of the lived experience worker*	Co‐ideation	
	Being involved as a team member among nonpeer workers from the initial stages of implementation can be validating for lived experience workers. It is important to include lived experience workers in identifying roles and responsibilities, outlining specific steps and milestones, and defining goals and measures for implementation success in advance.	Before implementing a lived experience worker role: − Creativity workshops and regular meetings with individuals with lived experience − Review statistics, existing practices, and existing research on lived experience roles and interventions − Steering groups inclusive of individuals with lived experience
Construct 2: Assessing needs *The degree to which the organization collects information about priorities, preferences, and needs of lived experience workers and service users to support the implementation of the lived experience worker*		
Construct 3: Assessing context *The degree to which an organization collects information to identify and appraise barriers and facilitators to implementation of the lived experience worker*		
Construct 4: Planning *The degree to which a scheme or method of behavior and tasks for implementing a lived experience workforce are developed in advance and the quality of those schemes or methods*		
Construct 5: Tailoring strategies *The degree to which the organization chooses and operationalizes implementation strategies to address barriers, leverage facilitators, and fit context*	Co‐creation	
	The lived experience worker is unique, so partnering with people with lived experience to implement a lived experience worker role can assist in the effective implementation process. For example, recruitment processes, induction systems, and training requirements may need to be adapted to onboard lived experience workers.	− Sessions with people with lived experience to understand differences in roles/responsibilities and how change can be integrated into routine delivery of services − Convene staff meetings or workshops with individuals with lived experience to document ideas and processes − People with lived experience as formally appointed implementation leaders (see Domain Four)
Construct 6: Engaging *Attracting and involving lived experience workers and service users in the integration of the lived experience role*		
Construct 7: Doing *Carrying out or accomplishing the introduction of a lived experience workforce according to plan*	Co‐implementation	
	The process of implementation must be iterative, given the changing nature of the roles and responsibilities of lived experience workers. As the role develops, training and other support (e.g., debriefs) may need to be introduced. The necessary training and support may vary between lived experience workers.	− Lived experience workers assist in identifying barriers to and enablers for organizational change − Execute procedures for data collection and analysis to ascertain the effectiveness of the integration of the lived experience role or intervention − Provide organizational protocols that support the integration of lived experience workers − Regular meetings between lived experience workers and other individuals with lived experience for ongoing review of the process − Staff training and development (nonpeer and lived experience worker)
Construct 8: Reflecting and evaluating *Quantitative and qualitative feedback about the progress and quality of implementation accompanied by regular personal team debriefing about progress and experience*	Co‐evaluation	
	Evaluation of the lived experience role is essential to the sustainability of the lived experience workforce. This evaluation must include establishing the effect of people with lived experience engaging in this role and how the organization can better support their lived experience workforce.	− Workshops with individuals with lived experience to consider/interpret findings − Modify existing or implement new data collection systems − Ongoing staff training and development in new ways of working (for both nonpeer and lived experience workers)
Construct 9: Adapting *Modify the innovation and/or the inner setting for optimal fit and integration into work processes*		

Abbreviation: CFIR, Consolidated Framework for Implementation Research.

### 3.7 Lived experience representation

The scoping review and co‐creation sessions highlighted, that for people with LEX to actively participate in the provision and enhancement of service delivery, there must be representation of the values of LEX within organizational policies and practices and representation of people with LEX across the organizational structure. Organizational structures lacking representation and recognition of LEX were reported as oppressive to LEX workers.^45^ This resulted in LEX workers feeling undervalued and impeded their ability to provide support.^46^ One of the major contributors to job dissatisfaction, disengagement, exhaustion, and turnover identified was the resistance from nonpeer workers,^47^ or the structural and interpersonal stigma that existed in non‐recovery‐oriented spaces.^32^, ^48^, ^49^, ^50^ Because staff members' attitudes toward LEX workers were reported as being influenced by agency policies and programmatic rules,^47^ clear policies and procedures were identified as necessary to provide adequate resource allocation and ongoing training to address workplace stigma.^32^ For example, additional training may be necessary for nonpeer workers in the values that underpin lived experience.^45^ Structural policies and processes, such as supervision and training, are included in the inner setting domain (Domain Three) (Table 2). These findings were further validated in the co‐creation session with people working alongside LEX workers, who requested further training to adequately support LEX workers in their roles.

The integration of LEX workers into an organization required the encouraged participation of LEX workers in staff meetings as well as organizational structures that allowed for LEX workers to collaborate with other staff members.^38^, ^42^, ^47^ Being a part of an interdisciplinary team was reported as a necessary component and positive experience related to the integration of LEX workers into an organization.^40^, ^47^, ^51^ Further, consultation and collaboration among LEX workers and other nonpeer staff was an effective way of addressing stigma in the workplace^10^ and assisted in the development and facilitation of effective programs and interventions.^42^ In work environments where LEX workers shared experiential knowledge, LEX workers were able to challenge the dominant ways of working and influence service delivery.^42^, ^43^, ^52^, ^53^ The individuals domain (Domain Four) includes the various roles and characteristics of individuals involved in the implementation process, and the characteristics of lived experience workers (Domain Five) describe the interplay between LEX workers and the organization (Table 2). Lived experience workers act as agents of person‐centered care, upholding values of recovery and agency amongst service users.^42^, ^52^ Thus, the representation of LEX within an organization in addition to the structural policies that uphold the values of LEX becomes the impetus toward person‐centered, integrated models of care.

## 4 DISCUSSION

Implementation science frameworks are increasingly used across a range of contexts to enhance the implementation of health innovations. In particular, the CFIR has been adapted for diverse innovations and settings to identify barriers and facilitators to implementation outcomes.^18^ Recent (2022) updates to the CFIR highlight the need for users to fully operationalize constructs by adapting and using language that is meaningful for the context and individuals involved in implementing and delivering the innovation.^18^ This study aimed to operationalize the constructs of the CFIR using a rigorous scoping review methodology,^21^ including co‐creation sessions, so it can be used by health services seeking to build and strengthen their LEX workforce. Although there may be additional considerations for discrete organizations or initiatives, this current study aimed to develop a usable implementation framework for organizations who want to begin integrating a LEX workforce. We recommend organizations wishing to use the framework to guide how they engage and support LEX workers conduct further co‐creation to ensure the applicability and usability of the framework within their local context.

Recent popularization and formalization of LEX workforces globally require robust, evidence‐based frameworks to ensure organizations can effectively introduce and support workers in these roles. The extended CFIR framework, CFIR‐LEX, can be used by researchers and service providers to ascertain the barriers and facilitators of involving LEX workers in health service provision, improve the integration of LEX workers into service delivery, ensure the safety and sustainability of workers, and expand the workforce to other crisis and recovery services. The domains of the CFIR‐LEX include the following: the characteristics of the innovation being implemented (Domain One); external economic, political, and social context (Domain Two); internal structural, political, and cultural context (Domain Three); roles and characteristics of those involved in the implementation process (Domain Four and Five); and the process by which implementation is accomplished (Domain Six) (refer to Tables 2 and 3). Using the framework to design service strategies to establish and grow the LEX workforce will ensure that a holistic and evidence‐informed approach is taken to increase the likelihood of positive outcomes.

The key components to the successful integration of a LEX worker in a health setting were: (1) an organizational commitment to engaging people with LEX in the co‐creation of the LEX role and how interventions could be delivered; and (2) organizational representation of people with LEX.

This framework was developed through a co‐creation process. Co‐creation is an ongoing collaborative process that allows LEX workers to develop and design their roles within the organization while representing the patient's voice in the co‐production of care. LEX workers involved in the co‐creation sessions described the importance of providing feedback to the organization about their experience in the role and what the service users need. This was valuable because it allowed them to influence and improve service delivery and promote integrated care. This finding suggests that some of the barriers experienced by LEX workers contributing their experiential knowledge to the service and influencing healthcare practices and policies may be addressed through co‐creation processes. Furthermore, the successful engagement of a LEX workforce within an organizational setting occurs when LEX exists at all levels to impact service quality in terms of accessibility and patient‐centeredness.^5^, ^11^, ^54^, ^55^ The LEX workers involved in the co‐creation sessions valued the presence of LEX across the organizational structure and the ability to link with other LEX workers for support. Healthcare systems increasingly rely on the contributions of service users to share information, competencies, and skills with service providers to co‐create value,^19^ and this study identified LEX workers as potential facilitators of this co‐creating partnership in direct service delivery.

This study highlights that despite the popularization and formalization of peer roles in health service delivery,^9^ LEX roles remain undervalued. Current healthcare policy is informed by public health research operating within the context of evidence‐based medicine, which places the views of service users below randomized controlled trials in the hierarchy of evidence.^56^ This may lead to the perception of the introduction of LEX roles in a health service as either tokenistic or unfounded. Challenging the biomedical model of care at an organizational level requires ongoing investment in training for LEX workers to gain experiential knowledge—the integration and translation of LEX^54^—and for nonpeer workers to understand the value of experiential knowledge in health service delivery. Ensuring a structured approach to the engagement of new workforces, with a framework to assess progress, is essential to achieving integrated systems of care.

### 4.1 Strengths and limitations

The multifaceted approach to the development of a framework, including a rigorous scoping review methodology and well‐established co‐creation process, was a key strength of this project. The co‐creation process enabled LEX workers and their nonpeer counterparts to validate the findings from the scoping review, build on available evidence, and contribute their expertise and perspective on the findings. Embedding LEX voices in the framework development process was a key mechanism to operationalizing the framework. However, there were some limitations. There are limitations associated with the time period of the review (2016–2022), such as excluding fields of health care where peer support has been naturalized (e.g., disability support^57^). Yet, this time period captures the dynamic state of LEX roles as reflected in the recency of international healthcare policies for the institutionalization of peer support in health services.^58^, ^59^ The co‐creation process was limited by the recruitment strategy, with only one organization represented, and only one session with LEX workers, and one with their nonpeer counterparts. We also did not collect demographic information, so it is possible that others would have similar and different views to those presented in these sessions. The organization had recently (within 2 years) established a LEX workforce, so we believe the co‐creation findings are applicable to other organizations planning to involve LEX workers. We recommend future researchers consider recruiting widely across various university and community networks and budgeting appropriately for planning and executing co‐creation sessions including compensation for people's time and efforts. We also point researchers to recent work on capturing and analyzing demographic data to ensure diversity of participation and transferability of findings.^60^ In light of these limitations, further research is required to corroborate the usefulness of this framework and the use of this framework in the context of people with LEX working in other roles such as administration, management, policy, research, and education.

## 5 CONCLUSION

The active engagement of people with LEX in the co‐creation process and representation of LEX across the various levels of an organization are the critical forces necessary to shift away from a traditional medical model of healthcare towards a person‐centered, integrated care model. As the state of the LEX workforce expands across healthcare systems, the operationalized CFIR for the context of LEX workers functions as a useful and evidence‐based tool for researchers to identify barriers and facilitators of the engagement of LEX workers and for service providers who wish to introduce a LEX workforce into their service model.

## AUTHOR CONTRIBUTIONS

**Alayna Carrandi**: Conceptualization; data curation; formal analysis; writing—original draft; methodology; investigation; project administration; writing—review and editing. **Yanan Hu**: Data curation; formal analysis; writing—review and editing; methodology; investigation; validation. **Katherine McGill**: Conceptualization; formal analysis; writing—review and editing; investigation; supervision; validation. **Sarah Wayland**: Conceptualization; formal analysis; investigation; supervision; writing—review and editing; validation. **Shae Karger**: Data curation; writing—review and editing; validation. **Myfanwy Maple**: Conceptualization; data curation; formal analysis; supervision; methodology; investigation; writing—review and editing; validation.

## CONFLICT OF INTEREST STATEMENT

The authors declare no conflict of interest.

## ETHICS STATEMENT

Ethics approval for the co‐design sessions was granted by the University of New England's Human Research Ethics Committee (Approval No. HE21‐224). People involved in the co‐creation sessions provided written, informed consent for their data to be included in the publication of results.

## Supporting information

Supporting information.

## Data Availability

The data from co‐creation sessions are available from the corresponding author upon reasonable request. The data are not publicly available due to privacy or ethical restrictions. Data sharing from the scoping review is not applicable, as no new data were created or analyzed.
