# Supplementary material for: Operationalizing the Consolidated Framework for Implementation Research to build and support the lived experience workforce in direct health service provision
Source: Health Expect. 2024 Apr 3;27(2):e14035. doi: 10.1111/hex.14035 (PMC10989156; doi:10.1111/hex.14035)
Supplement: Supplementary file 1 — Supporting information. [file HEX-27-e14035-s001.docx]

Appendix 1: Full search strategy

*Initial Search*

Database Platform/Interface: ProQuest

Database: PsycINFO and PsycArticles

Database date range: 1894 to present

Date database last searched or consulted: 16 July 2021

Years covered by the search: January 2016 – December 2021

Complete search strategy:

ab(("user experienc*" OR "public involvement" OR "patient involvement" OR "user perspective" OR "user led" OR "user-led" OR "expert panel" OR advisory OR "reference group" OR "patient centred" OR "patient-centred" OR "patient centered" OR "patient-centered" OR "patient led" OR "patient-led" OR "patient activat*" OR "peer led" OR "peer-led" OR consumer OR "lived experience*") ) AND ab("peer counsel*" OR "peer advocate" OR "consumer-provider" OR (peer NEAR/4 "health assistant") OR (peer NEAR/4 "case manager") OR "consumer advocate" OR (peer NEAR/4 provider*) OR "peer navigator" OR "peer work*" OR "peer specialist*" OR "peer consultant*" OR "peer educat*" OR "consumer surviv*" OR “expert by experience”) AND ab(provide* OR "service provider*" OR staff* OR team* OR personnel OR employ* OR "case manag*" OR "service delivery" OR collaborat* OR aide* OR specialist* OR consultant* OR delivered OR operated OR assisted OR led OR managed OR conducted OR directed OR run)

Returns/total results from search: 260

Database: Scopus

Database date range: 1957 to present

Date database last searched or consulted: 16 July 2021

Years covered by the search: January 2016 – December 2021

Complete search strategy:

ABS(("user experienc*" OR "public involvement" OR "patient involvement" OR "user perspective" OR "user led" OR "user-led" OR "expert panel" OR advisory OR "reference group" OR "patient centred" OR "patient-centred" OR "patient centered" OR "patient-centered" OR "patient led" OR "patient-led" OR "patient activat*" OR "peer led" OR "peer-led" OR consumer OR "lived experience*")) AND ABS("peer counsel*" OR "peer advocate" OR "consumer-provider" OR (peer NEAR/4 "health assistant") OR (peer NEAR/4 "case manager") OR "consumer advocate" OR (peer NEAR/4 provider*) OR "peer navigator" OR "peer work*" OR "peer specialist*" OR "peer consultant*" OR "peer educat*" OR "consumer surviv*" OR "expert by experience") AND ABS(provide* OR "service provider*" OR staff* OR team* OR personnel OR employ* OR "case manag*" OR "service delivery" OR collaborat* OR aide* OR specialist* OR consultant* OR delivered OR operated OR assisted OR led OR managed OR conducted OR directed OR run) AND ( LIMIT-TO ( PUBYEAR,2021) OR LIMIT-TO ( PUBYEAR,2020) OR LIMIT-TO ( PUBYEAR,2019) OR LIMIT-TO ( PUBYEAR,2018) OR LIMIT-TO ( PUBYEAR,2017) OR LIMIT-TO ( PUBYEAR,2016) OR LIMIT-TO ( PUBYEAR,2015) OR LIMIT-TO ( PUBYEAR,2014) OR LIMIT-TO ( PUBYEAR,2013) OR LIMIT-TO ( PUBYEAR,2012) OR LIMIT-TO ( PUBYEAR,2011) ) AND ( LIMIT-TO ( LANGUAGE,"English" ) )

Returns/total results from search: 545

Database: Informit

Database date range: 1965 to present

Date database last searched or consulted: 16 July 2021

Years covered by the search: January 2016 – December 2021

Complete search strategy:

[Abstract: 'user experienc*' OR Abstract: 'public involvement' OR Abstract: 'patient involvement' OR Abstract: 'user perspective' OR Abstract: 'user led' OR Abstract: 'user-led' OR Abstract: 'expert panel' OR Abstract: advisory OR Abstract: 'reference group' OR Abstract: 'patient centred' OR Abstract: 'patient-centred' OR Abstract: 'patient centered' OR Abstract: 'patient-centered' OR Abstract: 'patient led' OR Abstract: 'patient-led' OR Abstract: 'patient activat*' OR Abstract: 'peer led' OR Abstract: 'peer-led' OR Abstract: consumer OR Abstract: 'lived experience*'] AND [Abstract: 'peer counsel*' OR Abstract: 'peer advocate' OR Abstract: 'consumer-provider' OR [Abstract: peer near/4 AND Abstract: 'health assistant'] OR [Abstract: peer near/4 AND Abstract: 'case manager'] OR Abstract: 'consumer advocate' OR Abstract: peer near/4 provider* OR Abstract: 'peer navigator' OR Abstract: 'peer work*' OR Abstract: 'peer specialist*' OR Abstract: 'peer consultant*' OR Abstract: 'peer educat*' OR Abstract: 'consumer surviv*' OR Abstract: 'expert by experience'] AND [Abstract: provide* OR Abstract: 'service provider*' OR Abstract: staff* OR Abstract: team* OR Abstract: personnel OR Abstract: employ* OR Abstract: 'case manag*' OR Abstract: 'service delivery' OR Abstract: collaborat* OR Abstract: aide* OR Abstract: specialist* OR Abstract: consultant* OR Abstract: delivered OR Abstract: operated OR Abstract: assisted OR Abstract: led OR Abstract: managed OR Abstract: conducted OR Abstract: directed OR Abstract: run] AND Limit To: Peer Reviewed AND Publication Date: (07/01/2011 TO 07/31/2021) AND Language: English

Returns/total results from search: 2

*Rerun Search*

Database Platform/Interface: ProQuest

Database: PsycINFO and PsycArticles

Database date range: 1894 to present

Date database last searched or consulted: 27 September 2022

Years covered by the search: January 2021 – September 2022

Complete search strategy:

ab(("user experienc*" OR "public involvement" OR "patient involvement" OR "user perspective" OR "user led" OR "user-led" OR "expert panel" OR advisory OR "reference group" OR "patient centred" OR "patient-centred" OR "patient centered" OR "patient-centered" OR "patient led" OR "patient-led" OR "patient activat*" OR "peer led" OR "peer-led" OR consumer OR "lived experience*") ) AND ab("peer counsel*" OR "peer advocate" OR "consumer-provider" OR (peer NEAR/4 "health assistant") OR (peer NEAR/4 "case manager") OR "consumer advocate" OR (peer NEAR/4 provider*) OR "peer navigator" OR "peer work*" OR "peer specialist*" OR "peer consultant*" OR "peer educat*" OR "consumer surviv*" OR “expert by experience”) AND ab(provide* OR "service provider*" OR staff* OR team* OR personnel OR employ* OR "case manag*" OR "service delivery" OR collaborat* OR aide* OR specialist* OR consultant* OR delivered OR operated OR assisted OR led OR managed OR conducted OR directed OR run)

Returns/total results from search: 38

Database: Scopus

Database date range: 1957 to present

Date database last searched or consulted: 27 September 2022

Years covered by the search: January 2021 – September 2022

Complete search strategy:

ABS(("user experienc*" OR "public involvement" OR "patient involvement" OR "user perspective" OR "user led" OR "user-led" OR "expert panel" OR advisory OR "reference group" OR "patient centred" OR "patient-centred" OR "patient centered" OR "patient-centered" OR "patient led" OR "patient-led" OR "patient activat*" OR "peer led" OR "peer-led" OR consumer OR "lived experience*")) AND ABS("peer counsel*" OR "peer advocate" OR "consumer-provider" OR (peer NEAR/4 "health assistant") OR (peer NEAR/4 "case manager") OR "consumer advocate" OR (peer NEAR/4 provider*) OR "peer navigator" OR "peer work*" OR "peer specialist*" OR "peer consultant*" OR "peer educat*" OR "consumer surviv*" OR "expert by experience") AND ABS(provide* OR "service provider*" OR staff* OR team* OR personnel OR employ* OR "case manag*" OR "service delivery" OR collaborat* OR aide* OR specialist* OR consultant* OR delivered OR operated OR assisted OR led OR managed OR conducted OR directed OR run) AND ( LIMIT-TO ( PUBYEAR , 2022) AND ( LIMIT-TO ( LANGUAGE,"English" ) )

Returns/total results from search: 157

Database: Informit

Database date range: 1965 to present

Date database last searched or consulted: 27 September 2022

Years covered by the search: January 2021 – September 2022

Complete search strategy:

[Abstract: 'user experienc*' OR Abstract: 'public involvement' OR Abstract: 'patient involvement' OR Abstract: 'user perspective' OR Abstract: 'user led' OR Abstract: 'user-led' OR Abstract: 'expert panel' OR Abstract: advisory OR Abstract: 'reference group' OR Abstract: 'patient centred' OR Abstract: 'patient-centred' OR Abstract: 'patient centered' OR Abstract: 'patient-centered' OR Abstract: 'patient led' OR Abstract: 'patient-led' OR Abstract: 'patient activat*' OR Abstract: 'peer led' OR Abstract: 'peer-led' OR Abstract: consumer OR Abstract: 'lived experience*'] AND [Abstract: 'peer counsel*' OR Abstract: 'peer advocate' OR Abstract: 'consumer-provider' OR [Abstract: peer near/4 AND Abstract: 'health assistant'] OR [Abstract: peer near/4 AND Abstract: 'case manager'] OR Abstract: 'consumer advocate' OR Abstract: peer near/4 provider* OR Abstract: 'peer navigator' OR Abstract: 'peer work*' OR Abstract: 'peer specialist*' OR Abstract: 'peer consultant*' OR Abstract: 'peer educat*' OR Abstract: 'consumer surviv*' OR Abstract: 'expert by experience'] AND [Abstract: provide* OR Abstract: 'service provider*' OR Abstract: staff* OR Abstract: team* OR Abstract: personnel OR Abstract: employ* OR Abstract: 'case manag*' OR Abstract: 'service delivery' OR Abstract: collaborat* OR Abstract: aide* OR Abstract: specialist* OR Abstract: consultant* OR Abstract: delivered OR Abstract: operated OR Abstract: assisted OR Abstract: led OR Abstract: managed OR Abstract: conducted OR Abstract: directed OR Abstract: run] AND Limit To: Peer Reviewed AND Publication Date: (07/01/2021 TO 09/30/2022) AND Language: English

Returns/total results from search: 0

Appendix 2: Complete data extraction form

| Data Extraction Items |
| --- |
| Title |
| Authors |
| Country in which the study conducted |
| Year study was published |
| Aim of study |
| Study reports on:   - Effectiveness - Experiences - Both - Other |
| Perspective   - Patient/service user - Health professional or manager - Lived experience worker - Other |
| Area of study   - Mental health - Sexual health - Forensic medicine - Geriatrics - Cancer - Other |
| Study design   - Randomized controlled trial - Non-randomized controlled trial - Cohort study - Cross-sectional study - Case control study - Qualitative research - Prevalence study - Case series - Case report - Other |
| Sample size |
| Outcome/evaluation measures |
| Term used for lived experience worker |
| Definition of lived experience worker |
| Expectations of the lived experience worker’s role (e.g., training, qualifications during the hiring process, etc.) |
| Activities performed by lived experience worker |
| Lived experience worker's level of involvement (e.g., paid, volunteer, and if they were accompanied by a health professional) |
| Context in which the lived experience worker performs those activities (e.g., hospital, aged care home, etc.) |
| Results of the study |

Appendix 3: Explanatory text for the domains of the adapted Consolidated Framework for Implementation Research for Lived Experience Workers

Domain One: Innovation Domain

When lived experience workers engaged in generative activities and tailored the service to meet the unique needs of the service user and fit the local context, this resulted in a sense of agency experienced by the lived experience worker (1). Furthermore, instances where the lived experience worker defined their role and designed the intervention prior to implementation allowed the lived experience worker to positively redefine their identity as a person living with an illness, disability, or history of using a service and enhanced their social bonds, confidence, and resilience (2). More commonly, lived experience workers were recruited for the position by a research team, team of clinicians, or other non-peer staff within an organization (1, 3-6).

Lived experience workers were hired based on organizational criteria and their presentation to the people hiring them or among the service users (1, 7). Some of the subjective measures that were used during the interview process include: confidence in sharing their lived experience, excellent communication skills, and level of recovery (8-11). Objective hiring strategies included requiring lived experience workers to complete training delivered by an outside organization or the research team (4, 9, 12, 13). A lack of collaboration in the development of the lived experience role and the service they deliver hindered the lived experience workers from influencing service delivery, which ultimately undermined the value of their lived experience in the organization.

Both non-peer staff and service users perceived the lived experience worker to be highly beneficial to the service model—both a feasible and acceptable addition to treatment and care (4, 5, 14-18). Health professionals valued the unique contribution of lived experience, as a deeply empathetic approach (19-22). Lived experience workers were also valued for the affect-focus quality of their work and the influence of this on the health service model, by challenging the dominants forms of knowledge that favor objectivity (21, 23). Instances where lived experience workers and non-peers collaborated in the delivery of a program or intervention, service users found this to be complementary and conducive to social learning and a positive group atmosphere (6). Education groups delivered by lived experience workers, compared to delivery by clinical staff, allowed service users to learn coping skills and safety as a priority of treatment (13). Additionally, peer-delivered services were successfully provided in conjunction with virtual products (15-18) or services co-led by lived experience workers and non-peer staff (6, 10, 13). The addition of self-disclosure provided by a lived experience worker offered distinct value when compared with interactions between service users and other non-peer staff (6, 13).

The introduction of lived experience roles into health service organizations was deemed a promising strategy for creating services that are more likely to respond effectively to the needs of service users (24). Lived experience workers offered emotional support to service users who hesitated to participate or access treatment (25). Lived experience workers built relationships with those whom it may have otherwise been difficult to create an ongoing relationship that encouraged treatment adherence and help-seeking (25, 26). The relationship between lived experience workers and service users existed on an emotional level (versus a clinical level), which led to less reluctance of service users to talk openly about the condition and associated distress (27).

Domain Two: Outer Setting Domain

In the co-creation sessions, non-peer workers discussed barriers to the implementation of the lived experience workforce in their organization. First, they discussed delays in the program roll-out and recruitment of lived experience workers due to COVID-19-related restrictions. Second, they discussed difficulties filling the lived experience role in certain areas due to local attitudes and stigma towards identifying as someone with lived experience. Non-peer workers found that partnering with external organizations whose focus is training and supporting lived experience workers was very helpful in the implementation process.

External policies are emerging as the lived experience workforce expands. In the United States, for example, there has been a formalized certified peer support (CPS) training program. As a result of the training, CPS workers can provide Medicaid-billed mental health peer support services. One study (28) found the institutionalization of CPS training has resulted in limited entry into and retention within the workforce and compromised the relationship between peer workers and their clients by increasing power differentials. Institutionalized peer support was reported as less flexible and more formal than peer support services prior to institutionalization, with less emphasis on the needs and preferences of individual clients and more time spent on paperwork (28). CPS workers must meet pay structure requirements and complete paperwork to be compliant with Medicaid-reimbursable services. Adams (2020) proposes the increased institutionalization of lived experience workers may not lead to positive outcomes (28).

Domain Three: Inner Setting Domain

Studies identified necessary organizational shifts to promote the effective integration of lived experience workers, including the representation of the values of lived experience within organizational policies and practices. When organizational strategies allowed for thorough consultation process with individuals with lived experience, lived experience workers acted as agents of client-centredness and recovery (29). Consequently, lived experience workers upheld consumer-driven and recovery-oriented care, promoted agency amongst service users and influenced the service provision model, when possible (22, 29, 30). Effective organizational strategies suggested were a duty of care, a confidentiality code, and supervision (19). The presence of lived experience across a range of leadership domains was reported as optimal, and having a supervisor with lived experience was a positive experience for lived experience workers providing direct support (27, 31). In clinical settings, health professionals agree that lived experience workers should have equal status in their team and lived experience workers add value to their work (20).

Organizational structures that were reported as oppressive to lived experience or did not prioritize lived experience in the service delivery model resulted in lived experience workers feeling ineffective or undervalued (32). For example, the lack of organizational understanding and recognition of lived experience roles were expressed as unclear titles, lack of communication and expectation, lack of representation of lived experience in the organization, and a lack of role support and training (33). When organizational strategies repressed lived experience workers’ influence on service delivery, lived experience workers felt undervalued and unrecognized (19).

A challenge to the integration of lived experience workers included insufficient verbal communication with professionals and other lived experience workers, as well as a lack of supervision (34, 35). Barriers to sharing their lived experience included fear of rejection and lack of formal support (36). Where supervision existed, it was effective at tracking progress, measuring fidelity, identifying barriers to progression, identifying strategies to overcome barriers, and exploring linkages service users have made with other health and community services (3, 8, 37). Supervision was a way of providing practical support and mentorship to lived experience workers, as well as a way of providing clarification of roles and expectations (6, 38). Supervision de-risked the interaction between the lived experience worker and the client (22), but it was also a way to ensure any significant symptoms experienced by the lived experience worker was addressed to prevent potential relapse (8, 39). Lived experience workers benefited by being reminded to practice their own wellbeing skills (40).

The integration of lived experience into an organization required the encouraged participation of lived experience workers in staff meetings as well as organizational structures that allowed for lived experience workers to collaborate with other staff members (17, 22, 41). Lived experience workers represented the service users’ lifeworld and highlighted a strong affiliation with service users (11, 30). Oftentimes, lived experience workers represented the people they supported demographically (e.g., age, gender, ethnicity, income), geographically (i.e., live in the same community), and socially (e.g., have encountered similar barriers in their recovery journey) (8, 9, 42). Consequently, lived experience worker straddled the liminal space between a service user and a health professional. Not only do lived experience workers make valuable contributions to service users, but they can also add value to the work of health professionals (20). By avoiding the identification with either the health professional or service user, lived experience workers provided experiential knowledge to both parties (11, 23). They can contribute their experiential knowledge to the service and promote clinician uptake and fidelity of recovery-oriented practices (30, 40).

Linking a lived experience worker with another lived experience worker was effective in fostering accountability, confidence, hope, connection, and support (4, 22). This occurred amongst lived experience workers (i.e., a lived experience worker supervising another lived experience worker) (4, 16) or among a lived experience worker and person with lived experience at a managerial or administrative level (34). Access to supervision from a more senior person with lived experience was associated with more positive workplace experiences with respect to job satisfaction, engagement, fulfillment, and retention (32, 43).

Lived experience workers facilitated consumer-driven care by bringing experiential expertise to health service provision which was identified as distinct from the approach of formally trained health professionals (22). Experiential expertise enabled reciprocity and mutuality through the following methods: expressing shared knowledge of treatment and/or living with a health condition; role-modelling; and validating others’ lived experience (23, 44). The shared interactional space—not accessible to health professionals and other non-peer staff—was created through the alignment of a service user’s illness trajectory and the lived experience worker’s recovery journey (23). This relational approach was reported as complementary to the traditional medical approach within a clinical context and assisted clinicians in developing recovery goals for the service user and supporting them to reach these goals (2, 17, 22, 30, 45). The lived experience worker also provided appropriate referrals to clinical mental health services when necessary (22). The complementary and collaborative relationship between a lived experience worker and non-peer staff member enriches and deepens the service users experience by allowing each role to contribute their respective expertise (46).

Acting as agents of client-centredness and recovery—ambassadors of the values underpinning lived experience—lived experience workers promoted client autonomy in settings with lower recovery orientation and limited consumer choice (22, 29, 30). Organizations that were identified as not recovery-oriented, such as medical or clinical organization, required additional training for non-peer workers in the values that underpin lived experience (32). Vital to the effective collaboration between lived experience and non-peer staff is creating an agreed-upon structure with role descriptions, with the acknowledgment of their potential for development and change throughout the implementation process (46). In work environments where lived experience initiatives were featured and taught to non-peer staff, values of lived experience permeated and changed the organizational culture (24, 29, 30). Collaboration among lived experience workers and other non-peer staff further challenged the “them/us” mentality of some health professionals and was an effective way of addressing stigma in the workplace (31, 47). Establishing a foundation of trust between lived experience workers and non-peer staff may support each worker to feel confident within the boundaries of their own role (46).

The values underpinning lived experience workers (e.g., experiential knowledge) countered the dominant ways of working within a medical model of healthcare (21), which inevitably brought challenges. Even when organizations felt clear on the designated roles of lived experience workers, uncertainty and attempts to avoid stigma led to questioning the need for a designated lived experience role, resulting in diminished motivation to employ lived experience workers (31, 48). One of the major barriers to the expansion of the lived experience workforce reported was the resistance from non-peer workers (41), or the structural and interpersonal stigma that existed in non-recovery-oriented spaces (19, 49). Stigma has been identified more often among lower-status non-peer staff who have high work-task overlap with lived experience workers (e.g., case managers) compared with non-peer staff with higher education attainment and professional prestige (e.g., doctors) (28). Co-workers with higher educational attainment and professional prestige are often more willing to value lived experience and integrate lived experience workers into workplaces (28).

Being a part of an interdisciplinary team was reported as a necessary component and positive experience related to the integration of lived experience workers into an organization (6, 20, 38, 41). This includes attending weekly meetings, representing the organization externally, attending conferences and professional development workshops, and contributing to program planning and research (38). Correspondingly, a common negative aspect of a lived experience worker’s role was the attitudes of clinicians and workplace culture, which was interrelated with job dissatisfaction, disengagement, exhaustion, and turnover intention (43, 50). Navigating oppressive structures within the workplace was difficult and impeded on lived experience workers’ ability to support individuals (33). Because the attitudes of staff were reported as being influenced by agency policies and programmatic rules (29), clear policies and procedures were identified as necessary to provide adequate resource allocation and ongoing training and support to address workplace stigma (19).

Despite stakeholders’ mostly positive outlook regarding lived experience workers, the questioning of how recovered someone needs to be effective in a health service setting and the uncertainty of the lived experience workers’ value persists. Contesting the value of experiential knowledge limits the ability of lived experience workers to provide meaningful support and strengthens ‘objective’ forms of knowledge defined through professional institutions (i.e., clinical settings) (51). Lived experience workers must set their own role parameters, and these parameters must be distinguishable from non-peer roles (48). The lived experience workforce and organization would benefit from engaging in the co-development of the lived experience role, which may call for an increase in resources and training (52). Role negotiation, role definition, and open communication about changing roles and boundaries is vital to promoting collaborative relationships between lived experience workers and non-peer staff (46).

A foundation of trust and support, as well as discussions regarding role definition, are critical to establishing and sustaining collaborative working relationships between lived experience workers and non-peer staff (46). When considering compatibility in terms of the integration of a lived experience worker into an interdisciplinary working team, a key challenge was the unclear role definitions and overlap of duties with other non-peer staff (39, 53, 54). Lived experience workers viewed their experiential knowledge as distinctly different from health professionals and non-peer staff and valued the openness of non-peer staff to the insight of lived experience on the processes of treatment and care (11, 24). A lived experience worker’s role was distinguished from that of a health professional in that a lived experience worker prioritized self-directed growth, rather than meeting program-directed and treatment-directed outcomes which is the focus of health professionals (55). Lived experience workers refer service users to health professionals as needed and are an external support to assist service users to achieve recovery (52, 55). The relationship between lived experience workers and service users existed on an emotional level (versus a clinical level), which led to less reluctance of service users to talk openly about the condition and associated distress (27).

Although regarded as largely positive, clarity on the role of lived experience workers and the perceived value of lived experience roles varies among managers, health professionals, and lived experience workers (31). Lived experience workers must collaborate and consult with stakeholders to develop and facilitate effective programs and interventions (22). The sharing of experiential knowledge challenges traditional models of health service provision (21, 30). Stakeholders must prioritize the consultation and collaboration with lived experience workers through organizational representation of lived experience. For example, hiring only one lived experience worker in an organization limits the influence lived experience can have on a service model (41). Furthermore, having clear goals provided a sense of agency to lived experience workers and validates the lived experience value to the organization. Standardized reporting requirements, such as tracking goals, fostered helpful supervision to provide critical feedback, and identified additional training and education where necessary (8, 39). This helped to overcome the substantial barrier of role ambiguity and uncertainty.

Factors that must be in place to ensure the effective integration of lived experience roles includes adequate resources and communications and appropriate expectations (19, 34, 43). Job satisfaction and job retention among lived experience workers was reported as contingent upon proper resource allocation and ongoing support (34). The financial remuneration of lived experience workers varied from volunteer to paid positions (part-time and full-time) with employee benefits (e.g., employee assistance programs) (8, 24, 26, 38, 56). In instances where lived experience workers perceived the remuneration or hours allocated as insufficient, this was observed as a lack of recognition for their roles (24, 57), which led to the lived experience workers leaving their position for higher-paid and more stable roles (41). Job resources of social support, job control, feedback, rewards, and recognition are positive workplace experiences for lived experience workers, whereas low pay and lack of professional advancement are negative experiences (28, 43). Additionally, comprehensive training and ongoing supervision are needed to incur and sustain the integration (8, 58). Formal training was often how lived experience workers developed experiential knowledge to provide direct support in a manner that honored the values underpinning lived experience (28, 45). Training programs were commonly proctored by an external organization and addressed the following competencies: fundamentals of direct support; strategies for supporting recovery; understanding and navigating the health care systems; developing therapeutic relationships; supporting self-management; advocacy; and professionalism and ethics (4-6, 45, 56, 59-61). Another core element of training was disclosing lived experiences and constructing a personal story (5, 8, 45, 61).

In some instances, the development of a context-specific training program for lived experience workers was necessary to ensure the effectiveness of the intervention in improving health outcomes (62). In one study reporting on the introduction of a youth peer support worker (5), the research team had to develop an in-house training program at a later stage because the initial training model was inappropriate for the setting. This resulted in significant challenges including uncertainty amongst clinical staff about the role of the peer worker and a perceived sense of tokenism (5). A vital component to the integration of lived experience workers within organizations with non-peer staff is discussions from the outset around the negotiation, flexibility, and establishment of roles and role boundaries (46).

Domain Four: Individuals Domain

There was no available evidence in the peer-reviewed literature for the roles and characteristics of the following individuals: high-level leaders, mid-level leaders, opinion leaders, implementation facilitators, implementation leads, implementation team members, and other implementation support. These were explored in the co-creation sessions. Non-peer workers identified the context-specific implementation leaders, facilitators, team members, and support. Some of these individuals (e.g., program managers) were identified as playing more than one role in the implementation team. Program managers were individuals in the organization that had a formal or informal influence on the attitudes and beliefs of their colleagues with respect to integrating a lived experience workforce, and they were formally appointed with the responsibility of implementing a lived experience workforce and overseeing the work of lived experience workers. Funders and partner organizations were identified as external supports driving the expansion of the lived experience workforce. Non-peer workers also expressed the importance of involving clinical staff in the implementation process; however, they experienced a disconnect between lived experience workers and clinicians, which prevented them from working together.

Domain Five: Characteristics of the Lived Experience Workers Domain

Lived experience workers perceived their work to have unique value in terms of connecting with service users on an emotional level which promoted recovery, empowerment, and social connectedness (27, 36). Lived experience work fostered a sense of agency, redefined identity, confidence, and resilience for both service users and the lived experience workers themselves (1, 2, 8, 45). By reframing their lived experience as a professional resource, lived experience workers transition from a disempowered position—a patient needing help—to an empowered position—a professional who can provide help (11). Self-disclosure of lived experience was identified as an essential component of workers’ duties and was often one of the expectations of the role (6, 7, 44, 45). Through disclosure, lived experience workers challenged stigma and advocated for service users (27, 49). Self-disclosure was a source of inspiration and motivation for the service user by validating the service user’s experiences through mutual understanding (12, 19, 35).

Lived experience workers realized meaningful employment by using their story as an asset (11, 18, 63). Sharing experiences provided opportunities for self-discovery and personal development for both the lived experience worker and the service user (1, 44, 64). The effectiveness and sustainability of the lived experience role relied on the reciprocal identification with another’s lived experience (6, 63). Reciprocity was foundational to the work of lived experience workers and, coupled with the absence of power differential, distinguished their interactions with service users from other non-peer staff (12, 44). When lived experience workers construct a positive identity, they liberate themselves from restrictive role patterns and break down stigma and taboo (11, 49, 63). Providing support to others strengthened the lived experience workers’ ability to maintain personal recovery by keeping them connected to communities of support, providing opportunities to be of service, allowing them to pay forward what had been given to them, and a sense of accomplishment in being a part of the recovery of another individual (64).

Lived experience workers’ shift from being a service user to working in the recovery environment prompted several personal transitions that supported their own recovery, including building interpersonal relationships with community members and friends as well as changing health behaviors (38). This shift in identity was often a challenge for lived experience workers, as they found it difficult to separate their identity as a person with a health condition on a recovery journey who still required services from a person who provides health services (19, 25, 49). This manifested in difficulties reporting issues of triggering, reluctance to use health services, and feeling ‘stuck’ in positions that are dependent on the identity as a person with lived experience (26, 42). Thus, being a lived experience worker requires a reflexive practice (11). Further, training and guidelines on self-disclosure practices are essential (36). Refraining from overidentifying with service users and setting boundaries with service users was identified as essential to the lived experience role which must be supported by adequate organizational protocols (25, 44). Discussions of role boundaries and flexibility may bolster the lived experience worker’s confidence in their role (46).

Studies reported on the core components and peripheral components of a lived experience worker. Whereas core components are essential, periphery components refer to the elements and systems related to the intervention and organization that can be adapted to the setting without undermining the integrity of the intervention (65). The core component of a lived experience worker was performing direct support, such as individual support and group support, where the core values of lived experience were present (5, 26, 32, 38, 55, 57, 58, 63, 66). Some of the reported core values underpinning lived experience were self-determination, health and wellness, goal setting, hope, social inclusion, illness management, and stigma reduction (2, 4, 11, 41, 52, 56). Lived experience workers supplied support for service users when treatment decisions were being made and offered emotional support during a crisis, inpatient stay, or hospital discharge (8, 11, 12, 17, 19, 34). Other direct activities included setting and reaching goals, educating, and linking individuals to health resources (2, 19, 22, 32, 41, 55, 57). Peripheral components of a lived experience worker included non-direct tasks such as administrative work, teaching skills, and systems-level advocacy (5, 7, 17, 26, 27, 38, 41, 56, 57, 63). Lived experience workers reported spending work hours advocating at a community or organizational level for increased lived experience services and stigma mitigation (27, 41). Other peripheral duties included distributing supplies (e.g., meals), filling out paperwork, and navigating governmental policies and programs (19, 33, 34, 55).

The role of non-peer staff in health service provision comprised conveying educational and objective knowledge, while the role of the lived experience worker was to share lived experience, foster trust, and elicit hope (6). The lack of distinction between lived experience roles and non-peer roles may cause confusion. The distinction between case managers and lived experience workers was made where case managers focus on clinical care coordination and lived experience workers empower service users, promote service users’ educational growth, and support personal development (53). The overlap in duties include service users’ development, wellness and recovery, administrative tasks, and care coordination activities (53). The roles and competencies of a lived experience worker is also distinct from nurses. Lived experience workers focus on building up relationships that are supportive for both the service user and themselves and utilize their lived experience (39). Nurses are predominately focused on delivering instructions, being a team player, and ensuring security and control. Both nurses and lived experience worker assign a major role to the team in determining their satisfaction with their competencies (39).

Studies from the service user perspective reported service delivery by lived experience workers as a positive and powerful addition to service delivery (4, 13, 14, 19, 40, 42). Service users receiving support from people with lived experience benefited by feeling understood, hopeful, and less isolated (40). For example, Indigenous women taking part in a perinatal substance use program emphasized the value of accessing lived experience workers whose practice is grounded in their cultures and experiences (42). Lived experience workers facilitate recovery by providing practical and individualied support and social connection (42, 66, 67). These two components—providing practical support and understanding—were the implicit and explicit use of lived experience, respectively, in the role of a lived experience worker (35). These two components also facilitated the personal recovery for the lived experience worker (25).

Sharing lived experiences between lived experience workers and service users promotes engagement, hope, and belonging, whereas non-peer staffs’ input creates a sense of safety, structure, and purpose (68). Although the relationship between service user and non-peer staff was viewed positively, the non-peer approach to the service was perceived as task-oriented and focused on accomplishing concrete objectives (3). Sometimes, there were also power dynamics present, which was potentially harmful and sometimes reminded service users of previous negative experiences with health professionals (66). On the other hand, lived experience workers were described as process-oriented and flexible by individualizing the intervention to meet the needs of the service user (3). They built a safe space and a working partnership emphasizing hope and the possibility of change (35, 68). Service users were able to relate to the lived experience workers’ shared experience through a non-hierarchical relationship built by listening and trust (3). Lived experience workers were described by service users as role modeling a meaningful life, understanding the unique experience associated with the condition, and providing skills in the context of their own personal experiences (40). Additionally, carers perceived the service user’s relationship with a lived experience worker as positive (4, 67). They reported improvement in their own mood, confidence in recovery of their family member, and reduction in the carer burden (4, 67).

Domain Six: Implementation Process Domain

Implementation process is the final domain of the CFIR and consists of nine constructs. These refer to the essential activities and strategies used to implement an innovation in an organization. Although studies did not speak specifically to the stages at which the organizations introduced the lived experience workers, most studies engaged lived experience workers at the ‘doing’ stage. Lived experience workers were, for the most part, hired to deliver the intervention and not involved in the development of the role or intervention. The implementation process was further explored during the co-creation sessions with lived experience workers and their counterparts. Lived experience workers described being involved at the ‘doing’ stage and not being involved in creating their role. They expressed the importance of differentiating their role from other roles and the additional training they required to fulfill their role. The lived experience workers valued the presence of lived experience across the organizational structure and the ability to link with other lived experience workers to debrief and process their experience in the role. They described their role as dynamic and valued the pay increase they received when their position description expanded over the course of the implementation period. Being paid an adequate amount was key to feeling valued as a staff member. Lived experience workers discussed the issues of retention, turnover, and being under-staffed, and attributed this to the intensity of the role, highlighting importance of boundaries and self-care.

Lived experience workers expressed that service users did not always understand their role and were sometimes expecting a clinical solution. At times, the service users perceived the lived experience workers as volunteers, so they felt like they may have been burdening the lived experience workers. The lived experience workers attributed the misunderstanding to stigma and undervaluing lived experience. However, the lived experience workers did believe that service users appreciated them and the sense of validation and hope they received. Being able to give feedback to the organization about their experience in the role and what the service users need was important to lived experience workers. Different feedback methods included monthly check-ins with a peer mentor through an external organization, recurring feedback with manager, and optional debriefs following a session with service users. Some lived experience workers found various modes of feedback helpful. Overall, providing feedback and sensing that the feedback was taken on board was critically important to the lived experience workers.

References

1. O’Sullivan R, Hart W, Healy D. Transformative Rehabilitation: Exploring Prisoners’ Experiences of the Community Based Health and First Aid Programme in Ireland. Eur J Crim Pol Res. 2020;26(1):63-81.

2. Ahmadi Z, Sadeghi T, Loripoor M. The outcomes of peer-led diabetes education in comparison to education delivered by health professionals in Iranian patients. Health Educ Res. 2018;33(1):64-72.

3. Bochicchio L, Stefancic A, McTavish C, Tuda D, Cabassa LJ. “Being There” vs “Being Direct:” Perspectives of Persons with Serious Mental Illness on Receiving Support with Physical Health from Peer and Non-Peer Providers. Adm Policy Ment Health. 2021;48(3):539-50.

4. Fan Y, Ma N, Ma L, Zhang W, Xu W, Shi R, et al. Feasibility of peer support services among people with severe mental illness in China. BMC Psychiatry. 2019;19(1):360.

5. Hopkins L, Pedwell G, Wilson K, Howell-Jay P. Implementing youth peer support in an early psychosis program. Journal of Mental Health Training, Education and Practice. 2021;16(2):85-98.

6. Muralidharan A, Peeples AD, Hack SM, Fortuna KL, Klingaman EA, Stahl NF, et al. Peer and Non-Peer Co-Facilitation of a Health and Wellness Intervention for Adults with Serious Mental Illness. Psychiatr Q. 2021;92(2):431-42.

7. Sunguti JL, Tiam A, Masaba R, Waweru M, Kose J, Odionyi J, et al. Assessing treatment outcomes among peer educators living with HIV in Kenya. PLOS ONE. 2019;14(6):e0218774.

8. Conner KO, Gum A, Johnson A, Cadet T, Brown C. Peer Education: Productive Engagement for Older African Americans in Recovery From Depression. Gerontologist. 2018;58(5):813-24.

9. Conner KO, McKinnon SA, Roker R, Ward CJ, Brown C. Mitigating the stigma of mental illness among older adults living with depression: The benefit of contact with a peer educator. Stigma Health. 2018;3(2):93-101.

10. Dehghani A. A comparative study of the effect of peer-led and lecture-based education on health literacy in patients with multiple sclerosis. Int J Community Based Nurs Midwifery. 2021;9(1):76-85.

11. Kessing ML, Mik‐Meyer N. Negotiating mental illness across the lay‐professional divide: Role play in peer work consultations. Sociol Health Illn. 2022;44(4-5):815-29.

12. Balogun-Mwangi O, Rogers ES, Maru M, Magee C. Vocational Peer Support: Results of a Qualitative Study. J Behav Health Serv Res. 2019;46(3):450-63.

13. Crisanti AS, Reno J, Salvador JG, Killough C, Greene RN. Perceived helpfulness of peer-delivered trauma specific treatment: A randomized controlled trial. Psychol Serv. 2019;16(3):425-32.

14. Cook JA, Jonikas JA, Burke-Miller JK, Hamilton M, Powell IG, Tucker SJ, et al. Whole Health Action Management: A Randomized Controlled Trial of a Peer-Led Health Promotion Intervention. Psychiatr Serv. 2020;71(10):1039-46

15. Fortuna KL, Aschbrenner KA, Lohman MC, Brooks J, Salzer M, Walker R, et al. Smartphone Ownership, Use, and Willingness to Use Smartphones to Provide Peer-Delivered Services: Results from a National Online Survey. Psychiatr Q. 2018;89(4):947-56.

16. Fortuna KL, Storm M, Naslund JA, Chow P, Aschbrenner KA, Lohman MC, Bartels SJ. Certified Peer Specialists and Older Adults With Serious Mental Illness' Perspectives of the Impact of a Peer-Delivered and Technology-Supported Self-Management Intervention. J Nerv Ment Dis. 2018;206(11).

17. Gulliver A, Banfield M, Morse AR, Reynolds J, Miller S, Galati C. A Peer-Led Electronic Mental Health Recovery App in a Community-Based Public Mental Health Service: Pilot Trial. JMIR Form Res. 2019;3(2):e12550.

18. Üstel P, Smith MJ, Blajeski S, Johnson JM, Butler VG, Nicolia-Adkins J, et al. Acceptability and Feasibility of Peer Specialist-Delivered Virtual Reality Job Interview Training for Individuals with Serious Mental Illness: A Qualitative Study. J Technol Hum Serv. 2021;39(3):219-31.

19. Barr KR, Townsend ML, Grenyer BFS. Using peer workers with lived experience to support the treatment of borderline personality disorder: a qualitative study of consumer, carer and clinician perspectives. Borderline Personal Disord Emot Dysregul. 2020;7(1):20.

20. Chisholm J, Petrakis M. Perspectives about working alongside mental health peer workers in service teams. Advances in Mental Health. 2022;20(1):51-63.

21. Moore T, Zeeman L. More ‘milk’ than ‘psychology or tablets’: Mental health professionals’ perspectives on the value of peer support workers. Health Expect. 2021;24(2):234-42.

22. Van Zanden B, Bliokas V. Taking the next step: A qualitative study examining processes of change in a suicide prevention program incorporating peer-workers. Psychol Serv. 2022;19(3):508-18.

23. Oborn E, Barrett M, Gibson S, Gillard S. Knowledge and expertise in care practices: the role of the peer worker in mental health teams. Sociol Health Illn. 2019;41(7):1305-22.

24. Tseris E. The Expansion of the Peer Adviser Workforce: Opportunities and Challenges for Social Work. Australian Social Work. 2020;73(2):162-74.

25. Kido Y, Kayama M. Consumer providers' experiences of recovery and concerns as members of a psychiatric multidisciplinary outreach team: A qualitative descriptive study from the Japan Outreach Model Project 2011-2014. PLOS ONE. 2017;12(3):e0173330.

26. Wilson L, Vannice S, Hacksel C, Leonard L. Peer worker or client?: conflicting identities among peer workers engaged in harm reduction service delivery. Addict Res Theory. 2018;26(5):361-8.

27. Huisman A, van Bergen DD. Peer specialists in suicide prevention: Possibilities and pitfalls. Psychol Serv. 2019;16(3):372-80.

28. Adams WE. Unintended consequences of institutionalizing peer support work in mental healthcare. Soc Sci Med. 2020;262:113249.

29. Siantz E, Henwood B, Gilmer T. Peer Support in Full-Service Partnerships: A Multiple Case Study Analysis. Community Ment Health J. 2017;53(5):542-9.

30. Chisholm J, Petrakis M. Peer Worker Perspectives on Their Potential Role in the Success of Implementing Recovery-Oriented Practice in a Clinical Mental Health Setting. J Evid Based Soc Work. 2020;17(3):300-16.

31. Byrne L, Roennfeldt H, Davidson L, Miller R, Bellamy C. To Disclose or Not to Disclose? Peer Workers Impact on a Culture of Safe Disclosure for Mental Health Professionals With Lived Experience. Psychol Serv. 2022;19(1):9-18.

32. Clossey L, Gillen J, Frankel H, Hernandez J. The experience of certified peer specialists in mental health. Soc Work Ment Health. 2016;14(4):408-27.

33. Greer A, Buxton JA, Pauly B, Bungay V. Organizational support for frontline harm reduction and systems navigation work among workers with living and lived experience: qualitative findings from British Columbia, Canada. Harm Reduct J. 2021;18(1).

34. Mancini MA. An Exploration of Factors that Effect the Implementation of Peer Support Services in Community Mental Health Settings. Community Ment Health J. 2018;54(2):127-37.

35. Zeng G, Chung D. The stepped model of peer provision practice: capturing the dynamics of peer support work in action. The Journal of Mental Health Training, Education and Practice. 2019;14(2):106-18.

36. Walter O, Thomas EC, Salzer MS. Exploring Peer Specialists’ Experiences With Spirituality in Their Work: Recommendations for Future Directions. Psychiatr Rehabil J. 2021;45(1):95-102.

37. Cabassa LJ, Stefancic A, Lewis-Fernández R, Luchsinger J, Weinstein LC, Guo S, et al. Main outcomes of a peer-led healthy lifestyle intervention for people with serious mental illness in supportive housing. Psychiatr Serv. 2021;72(5):490-7.

38. Tookey P, Mason K, Broad J, Behm M, Bondy L, Powis J. From client to co-worker: a case study of the transition to peer work within a multi-disciplinary hepatitis c treatment team in Toronto, Canada. Harm Reduct J. 2018;15(1):41.

39. Debyser B, Duprez V, Beeckman D, Vandewalle J, Van Hecke A, Deproost E, Verhaeghe S. Mental health nurses and mental health peer workers: Self-perceptions of role-related clinical competences. Int J Ment Health Nurs. 2018;27(3):987-1001.

40. Barr KR, Townsend ML, Grenyer BFS. Peer support for consumers with borderline personality disorder: A qualitative study. Adv Ment Health. 2022;20(1):74-85.

41. Adams WE, Lincoln AK. Forensic peer specialists: Training, employment, and lived experience. Psychiatr Rehabil J. 2020;43(3):189-96.

42. Olding M, Cook A, Austin T, Boyd J. “They went down that road, and they get it”: A qualitative study of peer support worker roles within perinatal substance use programs. J Subst Abuse Treat. 2022;132.

43. Scanlan JN, Still M, Radican J, Henkel D, Heffernan T, Farrugia P, et al. Workplace experiences of mental health consumer peer workers in New South Wales, Australia: a survey study exploring job satisfaction, burnout and turnover intention. BMC Psychiatry. 2020;20(1):270.

44. Barrenger SL, Hamovitch EK, Rothman MR. Enacting lived experiences: Peer specialists with criminal justice histories. Psychiatr Rehabil J. 2019;42(1):9-16.

45. Jones M, Pietilä I. Personal perspectives on patient and public involvement – stories about becoming and being an expert by experience. Sociol Health Illn. 2020;42(4):809-24.

46. Hillman K, Pedlar D, Bibb J. My Space, Your Space, Our Space: Exploring the Potential of Collaborative Group Facilitation Between Therapists and Peer Workers in Mental Health Settings. Community Ment Health J. 2022;58(3):407-14.

47. Stanger Elran R, Hefer L. Opening a dialogue: lived experience meets Open Dialogue in Israeli mental health services. Adv Ment Health. 2022;20(2):146-56.

48. Roennfeldt H, Byrne L. How much ‘lived experience’ is enough? Understanding mental health lived experience work from a management perspective. Aust Health Rev. 2020;44(6):898-903.

49. Firmin RL, Luther L, Lysaker PH, Minor KS, McGrew JH, Cornwell MN, Salyers MP. Stigma resistance at the personal, peer, and public levels: A new conceptual model. Stigma Health. 2017;2:182-94.

50. Scanlan JN, Hancock N, Honey A. Evaluation of a peer-delivered, transitional and post-discharge support program following psychiatric hospitalisation. BMC Psychiatry. 2017;17(1):307.

51. Kirkegaard S. Experiential knowledge in mental health services: Analysing the enactment of expertise in peer support. Sociol Health Illn. 2022;44(2):508-24.

52. Kowalski MA. Mental Health Recovery: The Effectiveness of Peer Services in the Community. Community Ment Health J. 2020;56(3):568-80.

53. Crane DA, Lepicki T, Knudsen K. Unique and common elements of the role of peer support in the context of traditional mental health services. Psychiatr Rehabil J. 2016;39(3):282-8.

54. Hurley J, Cashin A, Mills J, Hutchinson M, Kozlowski D, Graham I. Qualitative study of peer workers within the ‘Partners in Recovery’ programme in regional Australia. Int J Ment Health Nurs. 2018;27(1):187-95..

55. Erangey J, Marvin C, Littman DM, Bender K, Mollica M, Milligan T, Lucas T. How do peer support workers value self‐directed growth over conventional change goals among young people experiencing homelessness? J Community Psychol. 2021.

56. Burr C, Rother K, Elhilali L, Winter A, Weidling K, Kozel B, Gurtner C. Peer support in Switzerland – Results from the first national survey. Int J Ment Health Nurs. 2020;29(2):212-23.

57. Cronise R, Teixeira C, Rogers ES, Harrington S. The peer support workforce: Results of a national survey. Psychiatr Rehabil J. 2016;39(3):211-21.

58. Tsai J, Klee A, Shea N, Lawless M, Payne KA, Goggin E, et al. Training peer specialists with mental illness in motivational interviewing: A pilot study. Psychiatr Rehabil J. 2017;40(4):354-60.

59. Chiocchi J, Lamph G, Slevin P, Fisher-Smith D, Sampson M. Can a carer (peer) led psychoeducation programme improve mental health carers well-being, reduce burden and enrich empowerment: a service evaluation study. The Journal of Mental Health Training, Education and Practice. 2019;14(2):131-40.

60. Druss BG, Singh M, von Esenwein SA, Glick GE, Tapscott S, Tucker SJ, et al. Peer-Led Self-Management of General Medical Conditions for Patients With Serious Mental Illnesses: A Randomized Trial. Psychiatr Serv. 2018;69(5):529-35.

61. Pfeiffer PN, Pope B, Houck M, Benn-Burton W, Zivin K, Ganoczy D, et al. Effectiveness of Peer-Supported Computer-Based CBT for Depression Among Veterans in Primary Care. Psychiatr Serv. 2020;71(3):256-62.

62. Sanders M, Tobin JN, Cassells A, Carroll J, Holder T, Thomas M, et al. Can a brief peer-led group training intervention improve health literacy in persons living with HIV? Results from a randomized controlled trial. Patient Educ Couns. 2021;104(5):1176-82.

63. Vandewalle J, Debyser B, Beeckman D, Vandecasteele T, Deproost E, Van Hecke A, Verhaeghe S. Constructing a positive identity: A qualitative study of the driving forces of peer workers in mental health-care systems. Int J Ment Health Nurs. 2018;27(1):378-89.

64. Scannell C. By helping others we help ourselves: insights from peer support workers in substance use recovery. Adv Ment Health. 2021.

65. Damschroder LJ, Aron DC, Keith RE, Kirsh SR, Alexander JA, Lowery JC. Fostering implementation of health services research findings into practice: a consolidated framework for advancing implementation science. Implement Sci. 2009;4(1):50.

66. Bardwell G, Kerr T, Boyd J, McNeil R. Characterizing peer roles in an overdose crisis: Preferences for peer workers in overdose response programs in emergency shelters. Drug Alcohol Depend. 2018;190:6-8

67. von Doussa H, Hegarty M, Sanders B, Cuff R, Tivendale K, McLean SA, Goodyear M. Peer support for children of parents with mental illness (COPMI) in Australia: responses from children, parents and facilitators of the CHAMPS peer support program. Adv Ment Health. 2022.

68. King AJ, Simmons MB. “The Best of Both Worlds”: Experiences of young people attending groups co-facilitated by peer workers and clinicians in a youth mental health service. Early Interv Psychiatry. 2022.
